# Differences in Social Hardships in Women and Men with Acute Myocardial Infarction: Impact on 30-Day Readmission

**DOI:** 10.1089/whr.2021.0150

**Published:** 2022-04-12

**Authors:** Faris Haddadin, Hassan Beydoun, Basera Sabharwal, Wojciech Rzechorzek, Mariam Khandaker, Alba Munoz Estrella, David Weininger, Bing Yue, Ricardo De La Villa, Jacqueline E. Tamis-Holland

**Affiliations:** ^1^ Section of Cardiology, Baylor College of Medicine, Houston, Texas, USA.; ^2^ Department of Cardiology, University of Arizona COM-Phoenix, Phoenix, Arizona, USA.; ^3^ Department of Cardiology, Icahn School of Medicine at Mount Sinai Morningside, New York, New York, USA.; ^4^ Department of Cardiovascular Medicine, Westchester Medical Center, New York Medical College, Valhalla, New York, USA.; ^5^ Department of Cardiology, University of Miami/Jackson Memorial Hospital, Miami, Florida, USA.; ^6^ Department of Cardiology, Mount Sinai Medical Center, Miami, Florida, USA.

**Keywords:** acute myocardial infarction, readmission, social hardships, Health Disparities

## Abstract

**Background:**

Studies have shown that women with acute myocardial infarction (AMI) have a higher prevalence of unfavorable social variables then men and have a worse outcome. Less is known regarding the impact of these social variables on 30-day readmission after AMI.

**Materials and Methods:**

We analyzed adult patients with AMI enrolled in a Quality Improvement Program intended to improve the peri-discharge care of patients with an AMI, and decrease all-cause 30-day unplanned readmissions. We compared clinical and social variables by gender. Multivariate logistic regression, with separate adjustment for clinical and for social variable, was used to measure adjusted odds for readmission by gender.

**Results:**

Among 208 patients included in our project 68 (32.7%) were women. Only 30.9% of women were married or had domestic partner at the time of the interview and only 16.2% were employed. Nearly half of women (48.5%) needed help with medical care, and 39.7% of women did not speak English as their first language. These variables were significantly different by gender. Rates of 30-day readmissions were higher in women than men (22.1% vs. 7.8%, *p* = 0.024). After adjusting for clinical variables this difference by gender in 30-day readmissions remained significant (odds ratio [OR] 3.34 95% confidence interval [CI] 1.1–11.1, *p* = 0.049). However, when adjusting for social variables, this difference was no longer noted (OR 0.87 95% CI 0.27–2.78, *p* = 0.822).

**Conclusion:**

Women with AMI are more likely than men to have unfavorable social factors that can impact recovery from AMI and women have a higher 30-day readmission rate. The higher 30-day readmissions in women appears to be influenced by these social factors. Health care interventions aimed at reducing 30-day readmission after AMI should focus on eliciting a detailed social history and providing aid for those requiring additional social support at home.

## Background

Cardiovascular disease (CVD) is the leading cause of death for women in the United States with about 40% of these deaths a result of coronary heart disease.^1^ Since 1984, more women have died of CVD each year than men.^2,3^ Although this gender disparity has been slowly narrowing over time, a gap continues to exist and women still have higher 30-day mortality after acute myocardial infarction (AMI).^2,4–7^ Differences in baseline comorbidities and presenting characteristics underlying pathophysiology contribute to the reported differences in outcomes.^8–11^ Women with AMI are less likely to receive revascularization as compared with men with similar presentation.^6,12^

In addition, several prior studies have reported that women with AMI face more social and economic barriers than men, which may hinder access to care and proper post-AMI follow-up.^13,14^ More recently, studies have shown that women with AMI also have higher all-cause 30-day readmission rates even after adjusting for demographic and comorbid clinical conditions.^15^ However, less is known regarding the impact of social variables on all-cause 30-day readmission after AMI in women versus men. To address this question, we examined clinical characteristics and social factors in women and men admitted with AMI at an academic hospital in New York City to determine the influence of such factors on 30-day readmissions after an AMI.

## Materials and Methods

### The Heart Liaison Program and patient population

This is a retrospective cohort study examining patients enrolled in the Mount Sinai Saint Luke's Heart Liaison Quality Improvement Program intended to improve the peri-discharge care of patients with an AMI, and decrease all-cause 30-day unplanned readmissions. In addition to standard care and counseling given by the patient's health care team, The Heart Liaison Program team physician provided adjunctive education and support (Table 1).

**Table 1.** Components of the Heart Liaison Program

Predischarge 30-minute meeting with the Heart Liaison Program physician team member including the following:
a. A detailed questionnaire/assessment of social and demographic information
b. An educational slide set outlining the risk factors leading to, and the diagnosis and treatment of an AMI
c. An adjunctive medication reconciliation
d. An opportunity for questions and answers
“Going Home After your Heart Attack” educational pamphlet
Scheduled 1-week follow-up appointment for high-risk patients and a 2-week appointment for all other patients
Postdischarge 48-hour phone call
24/7 (direct to doctor) patient “hot-line” for any questions, or concerns

AMI, acute myocardial infarction.

The program was initiated as a pilot feasibility project using internal medicine resident physician volunteers between June 2017 and April 2019, and the cohort examined was taken from patients enrolled during this time period. After 2018, this project was implemented as a hospital program coordinated by a team of nurse practitioners. This study, which is a retrospective evaluation using the AMI quality improvement database, was granted the ethical permission and clearance by the Institutional Review Board (IRB) of our health care system. All patients provided verbal consent to participate in the interview.

### Study variables and outcomes

Baseline clinical variables, hospital course and treatment, and social variables were recorded for each patient and were compared between women and men. Clinical variables included age, history of hypertension, diabetes mellitus, dyslipidemia, coronary artery disease, congestive heart failure, chronic kidney disease, smoking history, type of AMI, systolic dysfunction before discharge, type of AMI treatment, discharge medications, and high-risk “HOSPITAL” score (*i.e.*, HOSPITAL score >7).

The HOSPITAL score is a summative point-based scoring system that consists of seven clinical predictors with different weights, ranging from 0 to 13, designed to estimate the risk of 30-day readmission.^16,17^ The seven clinical variables of the HOSPITAL score are hemoglobin level <12 g/dL, discharge from oncology unit, sodium level <135 mEq/L, procedure performed during hospital stay, type of admission (urgent vs. elective), number of hospital admissions during the previous year and hospital stay ≥5 days.

Social factors collected during the predischarge interview included type of insurance, marital status or presence of a domestic partner, employment status, needing aid at home with medical care, presence of home health aide, being the primary caregiver to someone else, availability of an emergency contact person, education level, primary language spoken at home and during the interview, and availability of a transportation method to attend follow-up medical appointments. The main outcome of the study was all-cause 30-day readmission.

### Statistical analysis

Qualitative variables are presented as frequencies and percentages, whereas quantitative variables are presented as mean ± SD. Differences between groups were assessed with Pearson *X*^2^ for qualitative variables and Student's *t*-test for quantitative variables. Multiple logistic regression was used to estimate the all-cause 30-day readmission and its 95% confidence interval (CI).

Three modules were created to evaluate for the study outcome: crude unadjusted, adjusted only for clinical variables and adjusted only for social variables. Clinical variables in the second module included age, history of hypertension, diabetes mellitus, dyslipidemia, coronary artery disease, congestive heart failure, chronic kidney disease, smoking history, type of AMI, systolic dysfunction before discharge, AMI treatment, discharge medications, and high-risk hospital score (*i.e.*, >7).

Discharge medications included antiplatelet therapy, statins, beta-blockers, and Renin Angiotensin Aldosterone system inhibitors. Social variables in the third module included age, type of insurance, being married or having a domestic partner, employment status, requiring help with medical care, being caregiver to someone, having an emergency contact, level of education, primary language, requirement of translation during interview, availability of transportation to clinic appointments after discharge, and presence of family member or friend during interview. Age was included in the adjustment of both the clinical and the social module. A *p*-value of <0.05 was considered statistically significant. STATA (IC-15.1 version) was used for statistical analysis.

## Results

Between June 2017 and April 2019 we enrolled 208 eligible patients into our program. The mean age of enrolled patients was 64.7 ± 0.9 years and 68 (32.7%) were women. Table 2 outlines the baseline clinical variables and hospital course in women and men. Compared with men, women with AMI were significantly older and were more likely to have a history of hypertension. There were otherwise no significant differences by gender in baseline variables or treatment. Table 3 outlines the social variables in women versus men. As compared with men, a larger proportion of women had adverse social factors that might influence outcome.

**Table 2.** Baseline Clinical Characteristics Among Women and Men

Presenting clinical variables and treatment	Women (*n* = 68)	Men (*n* = 140)	*p*
Mean age	70.1 ± 1.6 years	62.1 ± 0.9 years	0.001
Race and ethnicity, *n* (%)			
Hispanic	30 (44.1)	48 (34.3)	0.110
Black	23 (30.8)	39 (27.8)	
White	13 (19.1)	45 (32.1)	
Asian	2 (6)	8 (5.8)	
Hypertension, *n* (%)	61 (89.7)	101 (72.1)	0.004
Diabetes mellitus, *n* (%)	31 (45.6)	52 (37.1)	0.234
Dyslipidemia, *n* (%)	42 (61.8)	88 (62.9)	0.879
History of coronary artery disease, *n* (%)	15 (22.1)	35 (25)	0.741
History of congestive heart failure, *n* (%)	24 (35.3)	45 (32.1)	0.651
Chronic kidney disease, *n* (%)	20 (29.4)	30 (21.4)	0.206
Smoking history, *n* (%)			
Current smoker	16 (23.5)	33 (23.6)	0.651
Former smoker	20 (29.4)	47 (33.6)	
Never smoker	32 (47.1)	60 (42.8)	
Type of AMI, *n* (%)			
STEMI	18 (26.5)	28 (20)	0.292
NSTEMI	50 (73.5)	112 (80)	
LVEF <40%, *n* (%)	21 (30.9)	50 (35.7)	0.491
AMI treatment, *n* (%)			
Percutaneous intervention	57 (83.8)	98 (70)	0.065
Coronary bypass surgery	5 (7.4)	27 (19.3)	
Medical treatment	6 (8.8)	14 (10.7)	
Discharge medications, *n* (%)			
Aspirin	63 (92.6)	133 (95)	0.495
P2Y12 inhibitor	61 (89.7)	134 (95.7)	0.093
Beta-blocker	63 (92.6)	131 (93.6)	0.803
Statin	68 (100)	139 (99.3)	0.485
ACE-I/ARB	42 (61.8)	79 (56.4)	0.464
High-risk HOSPITAL score (≥7)	3 (4.4)	13 (9.3)	0.216

ACE-I, angiotensin converting enzyme inhibitor; ARB, angiotensin receptor blocker; STEMI, ST elevation myocardial infarction; NSTEMI, non-ST elevation myocardial infarction.

**Table 3.** Baseline Social Variables Among Women and Men Infarction

Social variables	Women (*n* = 68)	Men (*n* = 140)	*p*
Insurance, *n* (%)			
Medicare	31 (45.5)	53 (37.8)	0.740
Medicaid	9 (13.2)	22 (15.7)	
Private insurance	25 (36.7)	57 (40.7)	
No insurance	3 (4.6)	8 (5.8)	
Married or had a domestic partner at the time of the interview, *n* (%)	21 (30.9)	79 (56.4)	< 0.001
Employed, *n* (%)	11 (16.2)	68 (48.6)	<0.001
Needed help at home with medical care, *n* (%)	33 (48.5)	31 (22.1)	<0.001
Primary caregiver to someone, *n* (%)	10 (14.7)	18 (12.9)	0.714
Had an emergency contact person, *n* (%)	44 (64.7)	97 (69.3)	0.507
Non-college educated, *n* (%)	48 (70.6)	79 (56.4)	0.049
Primary language not English, *n* (%)	27 (39.7)	36 (25.7)	0.039
Required translation, *n* (%)	17 (25)	28 (20)	0.411
Had transportation to attend appointments, *n* (%)	60 (88.2)	131 (93.6)	0.311
Family/friend present during interview, *n* (%)	27 (39.7)	50 (35.7)	0.576

A significantly larger proportion of women than men were readmitted within 30 days of discharge (22.1% vs. 7.8%, *p* = 0.024). Table 4 displays the reasons for admissions in women and men. The majority of women and men who were readmitted presented with a cardiovascular diagnosis. No deaths were reported in patients who were readmitted within 30 days. The univariate and multivariate odds for 30-day readmissions in women versus men are depicted in Table 5. After adjusting for medical factors, women were still more likely to be readmitted within 30 days of discharge. However, with adjustment of social factors, 30-day readmission in women and men were no longer significantly different.

**Table 4.** Reasons for Readmission Among Women and Men

Reason for readmission	Women (*n* = 15)	Men (*n* = 11)
All cardiovascular reasons	9/15 (60%)	8/11 (72.7%)
Acute myocardial infarction	3 (20%)	2 (22.2%)
Arrhythmia	3 (20%)	0
Acute heart failure exacerbation	1 (6.7%)	3 (33.3%)
Syncope or presyncope	2 (13.2%)	1 (11.1%)
Stroke	0	1 (11.1)
Pericarditis	0	1 (11.1%)
All noncardiovascular reasons	6/15 (40%)	3/11 (27.3%)
Gastrointestinal bleed	3 (20%)	1 (11.1%)
Noncardiac chest pain	1 (6.7%)	2 (22.2%)
Gastrointestinal track symptoms	1 (6.7%)	0
Acute kidney injury	1 (6.7%)	0

**Table 5.** Unadjusted and Adjusted All-Cause 30-Day Readmission in Women Compared with Men

Outcomes	Odds ratio (95% CI)	*p*-Value
Unadjusted all-cause 30-day readmission	2.76 (1.21–6.37)	0.017
Adjusted all-cause 30-day readmission for medical factors, age, and AMI type, treatment and discharge medications	3.34 (1.1–11.1)	0.049
Adjusted all-cause 30-day readmission for social factors, age, race, and ethnicity	0.87 (0.27–2.78)	0.822

CI, confidence interval.

## Discussion

Our study demonstrates that women with AMI were more likely than men to have adverse social factors, which have been shown to affect clinical outcomes. Rates of 30-day readmission were higher in women than men after AMI; however, this difference in 30-day readmissions by gender was no longer noted after adjusting for differences in social variables. All patients included in this study were given an equal opportunity to receive interventions known to improve the peri-discharge care of patients with AMI and reduce readmissions, including education, medication reconciliation, a 48-hour phone call, and early outpatient follow-up.

This study reflects a diverse patient population that mirrors the real demographic composition of New York City and the growing diversity of the United States. It sheds light on important and often forgotten social hardships in women suffering from an AMI. Our results were consistent with previous studies stipulating that women have more social barriers and that other factors, including health literacy, education, employment as well as social support can play a role in affecting outcomes in patients with AMI.^13,14,18–21^

In this study, a smaller proportion of women with AMI were married or had a domestic partner at the time of the interview, and a larger percentage of women than men needed help with medical care such as medication administration and timing of medications intake. Needing help with care could lead to lack of compliance with medication regimens, particularly if no one is available to supervise care. Patients living alone or without a spouse lack the added support that is often needed when recovering from a major illness; when in distress, these patients may not have anyone immediately available for support, therefore seeking medical care for issues that might have otherwise been addressed at home.^22^

We found a lower rate of English comprehension in women than men. Cultural and language barriers have been shown to negatively affect health literacy and adherence to the treatment plan when there are difficulties understanding the local language.^23^ It is possible that despite the fact that all of our patients were educated *via* a translator in their native language, non-English speaking patients still had difficulty understanding their disease process and as such a lower appreciation of the seriousness of their disease and importance of strict compliance with medications and follow-up.

In our study, women were less likely than men to have received a college education. A lower level of education has been associated with lower health literacy,^24^ and lower health literacy is associated with a higher rate of readmissions for acute coronary syndromes (ACS),^25^ and a higher 1-year mortality in patients admitted with CVDs.^26^ In addition, level of education achieved has been shown to influence rates of major adverse cardiac events in ACS patients.^27^

A systemic review that evaluated the influence of health literacy on the risk of coronary heart disease suggested that lower level of health literacy is associated with multiple obstacles toward lifestyle changes and the understanding of general disease knowledge.^19^ Patients with low health literacy are generally older, are less often employed, have lower educational levels and lower socioeconomic status.

Additional barriers to care that can lead to worse outcomes include lack of insurance or economic hardships as well as the inability to afford medications or clinic appointments or difficulty with transportation to hospital/clinics. Although our study did not report differences by gender in these variables, women were less likely than men to be employed, which can result in some financial hardships. Psychosocial risk factors differences between both genders have been shown to affect outcomes and presentation among patients with ACS.^13,14,28^

In an analysis of The Variation in Recovery: Role of Gender on Outcomes of Young AMI Patients (VIRGO) study the authors examined 3572 women and men between the ages of 18 and 55 years presenting with an AMI.^13^ Women were found to have a significantly higher level of perceived stress at baseline compared with men. A smaller proportion of women than men were married or living with a domestic partner, and fewer women than men were employed. Women were significantly more likely than men to have depression and report other life stressors, including major familial conflicts, severe illness/death in family members, and significant financial hardships.

In addition, more women than men were living in a household with children or grandchildren. After adjusting for baseline physical and psychosocial variables, the differences by gender in perceived stress was largely attenuated, although women still had a significantly higher baseline score. Recovery from the AMI at 1 month (with respect to angina status and perceived stress) was worse in women than men, but this difference was also largely attenuated after adjustment for baseline medical and psychosocial factors.

The findings in the VIRGO study are indirectly similar to our cohort. Women had more challenging social hardships that likely contributed to the higher readmission rate after discharge. The difference in readmission by gender was no longer significant after adjusting to social factors.

### Limitations

Our study was rather small in size, and this could have led to a lack of power to detect adjusted differences in 30-day readmissions. This is also reflected in the wide CIs of the adjusted and unadjusted readmission outcomes. For this reason, we cannot definitively conclude that gender is not a predictor of 30-day readmission after adjusting for the clinical variables. Finally, since this study was performed at a single hospital in an urban region, the population may not necessarily reflect the population of patients admitted to other medical centers in suburban or rural regions of the United States with a different demographic composition.

## Conclusion

Women with AMI are more likely than men to have unfavorable social factors that can impact recovery after AMI. Women have a higher 30-day readmission rate, which appeared to be largely influenced by these unfavorable social factors. Health care interventions aimed at reducing 30-day readmission after AMI should focus on obtaining a detailed interview to identify factors that may affect readmissions and when needed providing help for those requiring additional social support. Larger studies focusing on social determinants of health and gender disparities in women with AMI are needed to further solidify the need for such health care interventions and policies.

## Abbreviations Used

ACS: acute coronary syndromes
AMI: acute myocardial infarction
CI: confidence interval
CVD: cardiovascular disease
OR: odds ratio
VIRGO: The Variation in Recovery: Role of Gender on Outcomes of Young AMI Patients
